# Immediate Urethral Reconstruction for Membranous Urethral Injury During Rectal Surgery: A Case Report

**DOI:** 10.1002/iju5.70188

**Published:** 2026-05-12

**Authors:** Sou Kimura, Susumu Niikura, Yoshiyuki Ishiura

**Affiliations:** ^1^ Department of Urology Toyama Rosai Hospital Uozu Toyama Japan

**Keywords:** rectal surgery, urethral injury, urethral reconstruction

## Abstract

**Introduction:**

Urethral injury, although occurring at a measurable frequency during rectal surgery, remains underrecognized by urologists.

**Case Presentation:**

A 57‐year‐old man underwent laparoscopy‐assisted abdominoperineal resection for advanced rectoanal cancer. During anterior rectal dissection using electrocautery, the urethral catheter was inadvertently exposed. A partial tear of the dorsal half of the membranous urethra was directly visible through the perineal incision. The urethra was repaired in a watertight manner following standard principles of urethral reconstruction. On postoperative Day 15, retrograde urethrocystography confirmed the absence of extravasation or stricture, and the urethral catheter was removed. The patient subsequently demonstrated satisfactory voiding function.

**Conclusions:**

Immediate urethral reconstruction for injury sustained during rectal surgery can result in favorable postoperative outcomes. Awareness of this complication is essential for urologists, particularly as surgical techniques continue to evolve.

## Keynote Message

Immediate urethral reconstruction for membranous urethral injury during rectal surgery can achieve favorable postoperative outcomes. Urologists should remain vigilant about this potential complication.

## 1 Introduction

Although ureteral and bladder injuries are well‐recognized complications of colorectal surgery and are described in detail in some urology textbooks, reports of urethral injuries are scarce [1, 2]. In recent years, as newer rectal surgery techniques have become more widely adopted, urethral injury has emerged as a complication that occurs at a measurable frequency and requires careful attention during rectal surgery [3]. Nevertheless, this complication remains poorly recognized among urologists. We report a case of immediate urethral reconstruction performed for a urethral injury sustained during rectal surgery, resulting in favorable postoperative outcomes.

## 2 Case Presentation

**FIGURE 1:**
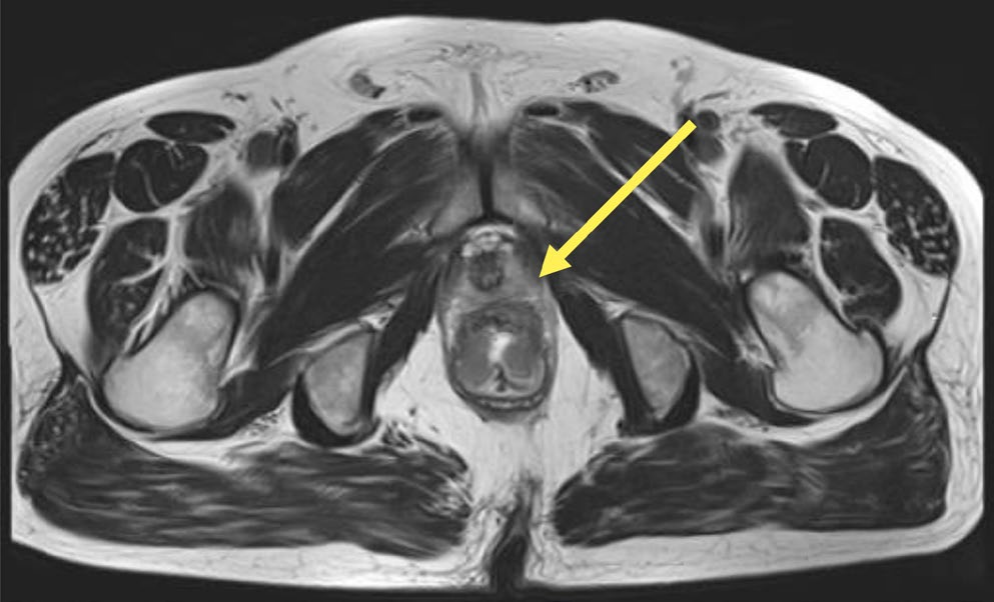
Preoperative MRI showing suspected extramural invasion of the anterior rectal wall (yellow arrow).

A 57‐year‐old man with no history of urological surgery or urinary catheterization was diagnosed with malignancy involving the rectum and anal canal. Magnetic Resonance Imaging (MRI) revealed suspected extramural invasion of the anterior rectal wall and multiple lymph node metastases (Figure 1), while Computed Tomography demonstrated multiple pulmonary and hepatic metastases. Based on these findings, a diagnosis of advanced rectal and anal canal cancer was made.

**FIGURE 2:**
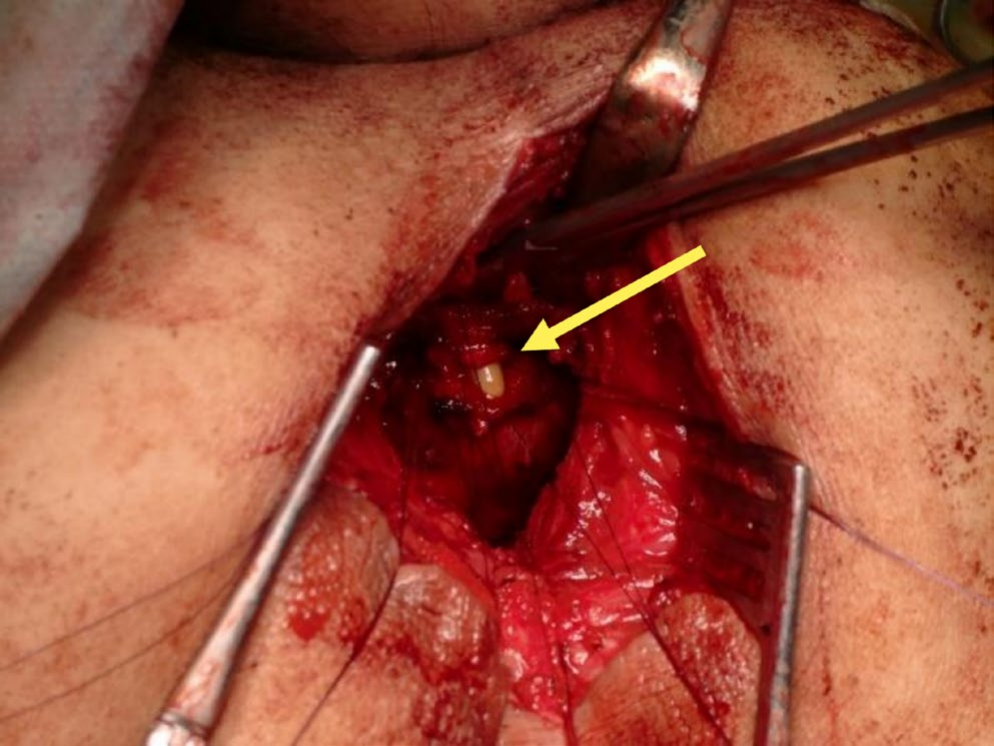
Intraoperative findings: Partial tear of the dorsal membranous urethra near the prostatic apex, exposing the urethral catheter (yellow arrow). The urethra was repaired with absorbable sutures.

Gastrointestinal surgeons at our hospital performed a laparoscopy‐assisted abdominoperineal resection (APR). During anterior rectal wall dissection through a perineal incision using an electrosurgical scalpel, the urethral catheter was inadvertently exposed (Figure 2). The surgeons concluded that the urethral injury had been caused by electrocautery. A urologist (Y.I.) was consulted intraoperatively and participated in the repair. The urethral injury was directly visible through the perineal incision and measured approximately 10 mm in length. The urethral injury involved a semicircumferential defect on its dorsal aspect and the anatomical relationship to the urethral sphincter was unclear. Because the injured tissue was minimally damaged, no trimming was required.

The urethra was repaired using five interrupted sutures using 3–0 Monocryl on a strong curved needle (26 mm, 5/8 circle) in a single layer, including the urethral mucosa. The repair followed standard reconstructive techniques: the needle was held vertically with a needle holder, a metal bougie was inserted into the urethral lumen, and the needle was passed through both mucosal edges. Watertight closure was confirmed by injecting saline through the external urethral meatus.

**FIGURE 3:**
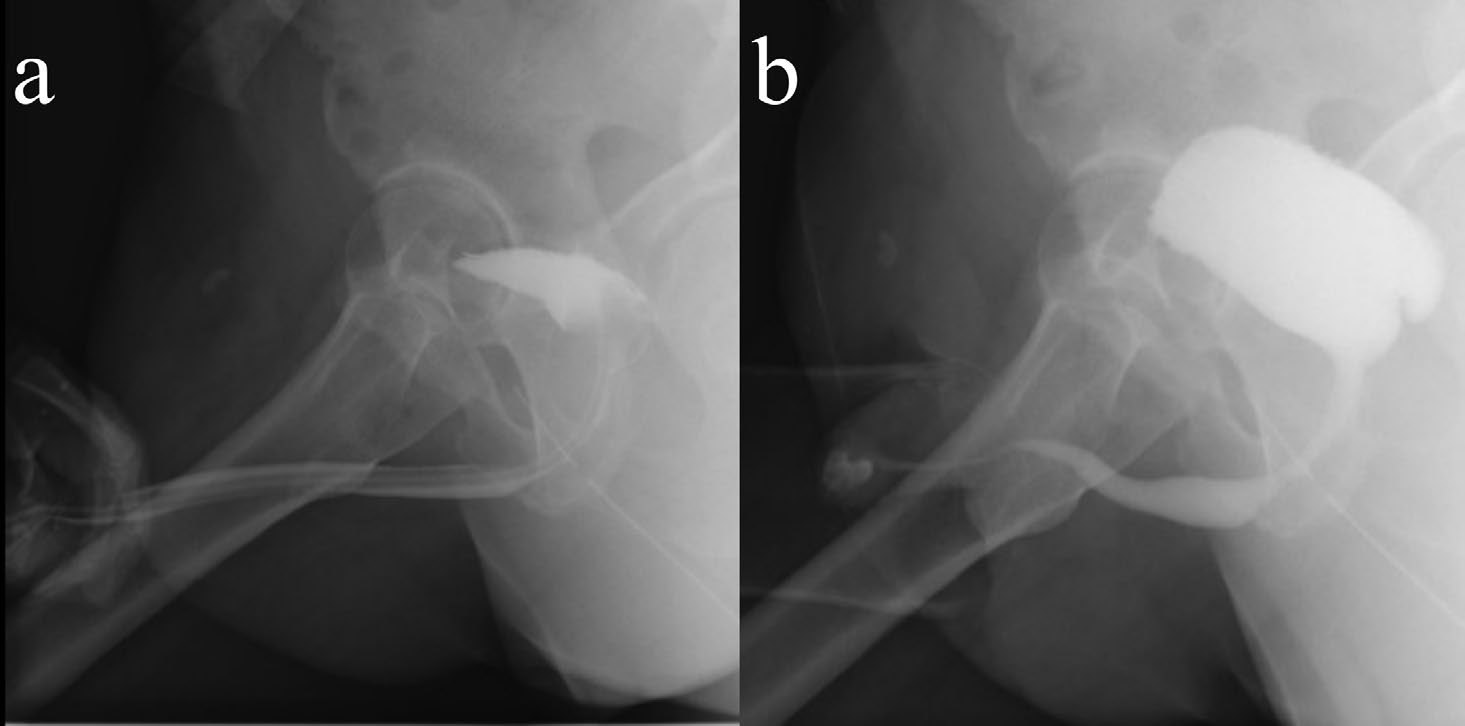
Postoperative Day 15: (a) RUG and (b) VCUG showing no extravasation or urethral stricture.

**FIGURE 4:**
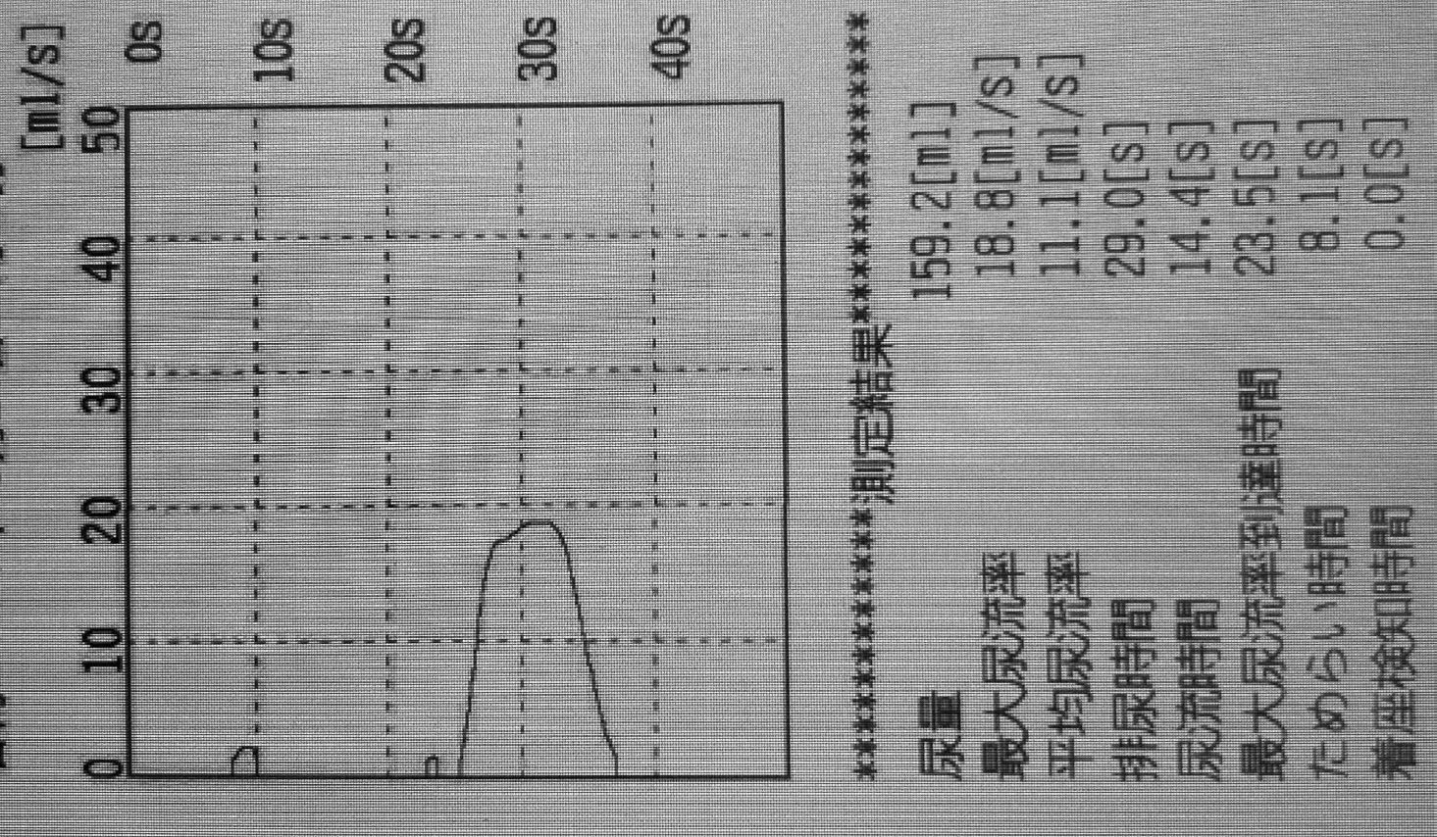
Five months postoperatively, uroflowmetry demonstrated a regular urinary flow pattern. Voiding volume: 159.2 mL; maximum urine flow rate: 18.8 mL/s; average urine flow rate: 11.1 mL/s.

On postoperative Day 15, retrograde urethrocystography (RUG) showed no extravasation or urethral stricture, and the urethral catheter was removed. Voiding cystourethrography (VCUG) confirmed the absence of urethral stricture during urination (Figure 3a,b). Following catheter removal, the patient had a strong urinary stream with no post‐void residual. Postoperative urinalysis revealed neither pyuria nor hematuria. At 5 months postoperatively, a 17 Fr flexible cystoscope could be passed without difficulty, and no anastomotic stricture was observed. Uroflowmetry and the International Prostate Symptom Score demonstrated satisfactory outcomes (Figure 4). At 4 years postoperatively, he has remained free of decreased urinary stream and urinary incontinence to date.

## 3 Discussion

APR is the standard surgical procedure for lower rectal cancer when anal preservation is not feasible. In contrast, transanal total mesorectal excision (TaTME), first reported in 2010 as an anal‐preserving technique, has rapidly gained popularity worldwide [3]. Both procedures are associated with urethral injury at a measurable frequency [4, 5]. Previous reports indicate that the incidence of urethral injury is 0.1%–2% in APR [6, 7] and 0.8% in TaTME [7]. Because urological injuries, including urethral injuries, tend to occur more frequently during the early learning curve of TaTME [3], the actual incidence in the current era—when TaTME is increasingly widespread—may be higher than previously reported.

In a series of 34 cases of urethral injury during TaTME [4], 32 injuries were recognized intraoperatively, and immediate repair was performed in 31 cases. Of the 34 urethral injuries, nine developed urethral complications, with a repair failure rate of up to 30%. Although urethral injury during rectal surgery is not uncommon and may lead to permanent complications, few reports have been authored by urologists [8], and almost no publications have described appropriate surgical management leading to favorable voiding outcomes.

The pathophysiological basis of urethral injury during rectal surgery lies in the close anatomical relationship between the rectum and the urethra, particularly the membranous urethra. The prostate gland is embryologically enclosed within the same fascia as the rectum, and membranous urethral injury may occur due to difficulty in identifying the correct dissection plane in the rectourethral muscle between the anterior rectal wall and the urethra. Anatomical studies indicate that the distance between the rectum and the membranous urethra is extremely short, ranging from 0.2 to 2.3 cm [6], emphasizing that the possibility of urethral injury during rectal surgery should always be considered. In women, the anterior surface of the rectum corresponds to the vaginal wall; therefore, urethral injury associated with rectal surgery is a male‐specific complication. The membranous urethra plays a pivotal role in urinary continence, and injury to this segment is therefore presumed to increase the risk of urinary incontinence. In the present case, because we were able to complete the anastomosis without tension using interrupted sutures, mobilization by distal dissection of the membranous urethra was not required. This likely contributed to preservation of urethral sphincter function and resulted in good continence. If significant anastomotic tension is anticipated to the extent that dissection and mobilization of the membranous urethra would be required, it may be prudent to consider limiting the initial procedure to suprapubic cystostomy and planning a delayed excision and primary anastomosis (EPA).

Interposition of vascularized tissue between the urethra and rectum may be considered in selected cases to reduce the risk of rectourethral fistula [9]. However, such risk is generally of greater concern when rectal anastomosis is performed. In this case, APR was performed without rectal anastomosis, and the risk of rectourethral fistula was considered low. For this reason, no interposition flap was used.

Regarding urethral injury, the EAU guidelines recommended deferred repair for urethral injuries associated with pelvic fracture; however in pelvic fracture settings, hemodynamic instability is common, and prioritizing urinary drainage is often necessary. Similarly, the EAU guidelines for ureteral injury state that immediate repair is preferred over delayed repair when the patient's general condition is stable [10].

It is difficult to draw definitive conclusions at present, but this is an important clinical issue that warrants further investigation. To our knowledge, this is the first report of immediate urethral reconstruction for a urethral injury sustained during rectal surgery, performed according to standard reconstructive principles and resulting in satisfactory voiding function for more than 5 months.

## 4 Conclusion

Immediate urethral reconstruction for membranous urethral injury sustained during rectal surgery can result in favorable postoperative outcomes. Urologists should recognize urethral injury as an important complication of rectal surgery, particularly in the context of modern surgical techniques.

## Ethics Statement

The authors have nothing to report.

## Consent

The authors have nothing to report.

## Conflicts of Interest

The authors declare no conflicts of interest.

## Data Availability

The data that support the findings of this study are available on request from the corresponding author. The data are not publicly available due to privacy or ethical restrictions.
